# Future Directions for Meningitis Surveillance and Vaccine Evaluation in the Meningitis Belt of Sub-Saharan Africa

**DOI:** 10.1093/infdis/jiz421

**Published:** 2019-10-31

**Authors:** Ryan T Novak, Olivier Ronveaux, André F Bita, Honoré Flavien Aké, Fernanda C Lessa, Xin Wang, Ado M Bwaka, LeAnne M Fox

**Affiliations:** 1 National Center for Immunization and Respiratory Diseases, Centers for Disease Control and Prevention, Atlanta, Georgia; 2 WHO Infectious Hazard Management, Geneva, Switzerland; 3 WHO Regional Office for Africa, Brazzaville, Congo; 4 Davycas International, Ouagadougou, Burkina Faso; 5 WHO Inter-Country Support Team West Africa, Ouagadougou, Burkina Faso

**Keywords:** Africa, meningitis epidemics, meningococcal, NmC, surveillance

## Abstract

In sub-Saharan Africa, bacterial meningitis remains a significant public health problem, especially in the countries of the meningitis belt, where *Neisseria meningitidis* serogroup A historically caused large-scale epidemics. In 2014, MenAfriNet was established as a consortium of partners supporting strategic implementation of case-based meningitis surveillance to monitor meningitis epidemiology and impact of meningococcal serogroup A conjugate vaccine (MACV). MenAfriNet improved data quality through use of standardized tools, procedures, and laboratory diagnostics. MenAfriNet surveillance and study data provided evidence of ongoing MACV impact, characterized the burden of non-serogroup A meningococcal disease (including the emergence of a new epidemic clone of serogroup C), and documented the impact of pneumococcal conjugate vaccine. New vaccines and schedules have been proposed for future implementation to address the remaining burden of meningitis. To support the goals of “Defeating Meningitis by 2030,” MenAfriNet will continue to strengthen surveillance and support research and modeling to monitor the impact of these programs on meningitis burden in sub-Saharan Africa.

For more than a century, meningitis epidemics have plagued the 26-country region of sub-Saharan Africa known as the “meningitis belt.” This region experiences high endemic rates of disease, with annual risk of epidemics during the January to June dry season, and periodic large regional epidemics occurring every 5–12 years. Before 2010, approximately 90% of meningitis cases during these epidemics were caused by *Neisseria meningitidis* serogroup A (NmA) [1, 2]. In 2008, Ministers of Health from the 26 meningitis belt countries signed the Yaoundé Declaration to eliminate meningococcal A epidemics as a public health concern in Africa [3]. More than 300 million people in 22 countries have been vaccinated with the meningococcal serogroup A conjugate vaccine ([MACV] MenAfriVac) since it was first introduced in 2010, and several studies have documented substantial immediate impact of mass MACV vaccination campaigns on disease and oropharyngeal carriage of meningococci [2, 4–7]. Nevertheless, to fully realize the Yaoundé Declaration’s goal to eliminate epidemics due to serogroup A meningococcus, further data and continued surveillance were required to evaluate the long-term effectiveness of MACV across the belt.

MenAfriNet was established in 2014 as a consortium of partners to support improved meningitis surveillance and provide a research platform in strategic, high-risk, meningitis belt countries to generate quality data to inform immunization policy and vaccine evaluation [8, 9]. Building upon a long history of international collaboration to strengthen the prevention, detection, and response to meningitis epidemics in Africa, MenAfriNet was led and implemented by African Ministries of Health, Agence de Médecine Préventive, the US Centers for Disease Control and Prevention (CDC), and the World Health Organization (WHO), and the consortium structure provided a framework to engage and collaborate with more than 30 international and nongovernmental organizations. The MenAfriNet consortium implemented population-based, case-based meningitis surveillance (CBS) with laboratory confirmation in 5 African meningitis belt countries (Burkina Faso, Chad, Mali, Niger, and Togo). The CBS was subsequently used to evaluate meningitis vaccines and conduct research to inform the need for revaccination, age group prioritization, and to monitor trends in meningitis due to other meningococcal serogroups and pathogens.

Country ownership of MenAfriNet activities was emphasized from the beginning of the program to encourage sustainable surveillance [8]. Annually, countries reviewed surveillance performance, developed work-plans to address performance gaps, and managed budgets for direct funding to support planned activities that complement and build on existing country systems. This emphasis on country ownership, supported by a robust consortium of diverse partners, ensured that CBS activities continued as planned despite numerous regional challenges, such as the 2014–2016 Ebola epidemic, terrorism events, and country insecurity/instability. One example of MenAfriNet country ownership is evident in the annual expansion of CBS from 2014 to 2018 despite no additional funding. Initially implemented in 76 districts across 4 countries, by 2018, Burkina Faso, Niger, Mali, Togo, and Chad expanded surveillance to 146 districts representing 48 million persons or 57% of the national population in these 5 high-risk countries [10]. MenAfriNet’s focus on country ownership has helped to achieve sustainable meningococcal surveillance in these countries.

Five years after the establishment of MenAfriNet, this journal supplement provides an opportunity to describe the surveillance strategy, review performance, and highlight successes and challenges. Papers elsewhere in this supplement detail how MenAfriNet has improved surveillance performance and strengthened country capacity for laboratory confirmation [10–16], contributed to a greater understanding of current meningitis epidemiology in the meningitis belt [17, 18], and provided a platform for vaccine evaluation and research to inform existing and future bacterial meningitis vaccine policies [19–21]. This paper presents a look forward at priorities to control meningitis due to pathogens other than NmA, based on findings from MenAfriNet and other surveillance and research in the region, and at future directions for continuing MenAfriNet to ensure quality data are readily available to inform and evaluate future vaccination strategies for the meningitis belt in sub-Saharan Africa.

## ONGOING DEVELOPMENTS IN MENINGOCOCCAL DISEASE AND ROLE OF MENAFRINET

To achieve the goal of eliminating NmA epidemics, the WHO recommended mass vaccination campaigns targeting persons aged 1–29 years (greater than 90% of the at-risk population) to rapidly achieve population immunity, followed within 1–5 years by MACV introduction into the routine childhood Expanded Programme on Immunization (EPI) at ages 9–18 months to sustain population-level immunity and ensure long-term suppression of disease [22]. An analysis of 2010–2015 surveillance data from 9 meningitis belt countries showed a 99% reduction in NmA disease incidence and a 57% reduction in overall incidence of suspected meningitis in areas that had implemented MACV [23]. Now, approximately 9 years after MACV introduction, MenAfriNet surveillance data, complemented by meningococcal carriage studies, have demonstrated substantial sustained impact of mass vaccination campaigns and routine immunization with MACV on NmA carriage and disease. Since 2014, only 5 sporadic cases of NmA disease have been confirmed in MenAfriNet countries—all in Burkina Faso before integration of MACV into routine EPI in 2017 [18, 24]. Recent modeling data suggest that immunity induced by routine childhood EPI vaccination with MACV confers >50% protection for 20 years in fully vaccinated populations [25]. Nevertheless, delays in full implementation of MACV rollout and integration into EPI, as well as potential waning immunity, pose risks for a resurgence of NmA disease and epidemics [6, 7].

Despite MACV success, a substantial burden of disease and risk of epidemics caused by *N meningitidis* serogroups other than NmA remain in the 26 sub-Saharan meningitis belt countries [17]. In 2013, a new molecular sequence type (ST) 10217 of meningococcal serogroup C (NmC) emerged in Nigeria [26]. Between 2013 and 2017, this unique clone spread to Niger and across the Northern States of Nigeria, causing more than 30 000 cases and 2500 deaths in 2017 [21, 27–30]. Attack rates observed during these NmC epidemics were comparable to those of historic NmA epidemics, with close to 1% of a district population affected in a given week (unpublished CDC data from 2015 Niger and 2017 Nigeria NmC outbreaks) [17]. In 2017, cases of NmC disease also occurred in other countries, both within (Benin, Burkina Faso, Mali) and outside (Liberia) the meningitis belt [17, 31–33]. MenAfriNet-supported investigations have documented the largest global outbreak of NmC in Niger, characterized the molecular ST of NmC strains from outbreaks in Mali and Burkina Faso, and documented substantial burdens of W and X in Togo and Chad, respectively [13, 14, 16, 18, 34]. In 2015 and 2017, a committee of experts was convened by WHO to review the emergence of NmC [35, 36]. The expert committee concluded that risk of NmC epidemics was likely to persist because of low-level population serogroup C immunity, and it noted that an ongoing risk of epidemics caused by serogroups X and W (NmX and NmW, respectively) also remains across the meningitis belt.

In addition to the remaining burden of non-A meningococcal meningitis, pneumococcal infection is now well documented to cause a significant proportion of the meningitis burden in the region [18]. Furthermore, outbreaks of pneumococcal meningitis have been reported in the meningitis belt of sub-Saharan Africa pre- and postintroduction of pneumococcal conjugate vaccines (PCVs) [37–41]. In 2015, Ghana reported the largest outbreak of pneumococcal meningitis in the meningitis belt post-PCV introduction, with a case-fatality ratio as high as 30%. This outbreak, which was caused by vaccine-type (serotype 1) pneumococci 3 years after introduction of 13-valent PCV into Ghana’s routine infant immunization program, has raised concerns about ongoing serotype 1 pneumococcal disease in meningitis-belt countries despite PCV introduction [40, 41].

The emergence of a unique epidemic-prone NmC strain, continued epidemic threat of NmW and NmX, ongoing endemic disease with focal outbreaks of pneumococcal meningitis, and continued occurrence of serotype 1 pneumococcal disease after implementation of infant PCV immunization underscore the value of high-quality meningitis surveillance, including strong laboratory capacity for rapid detection and serogrouping/serotyping of vaccine-preventable bacterial meningitis pathogens, and genome sequencing to monitor the molecular evolution of meningococcal and pneumococcal strains [20, 34, 42].

## THE FUTURE

To counter these ongoing challenges, new vaccines are in development and strategies for their utilization are being considered [9]. A number of multivalent conjugate meningococcal vaccines, including a pentavalent vaccine against serogroups A, C, W, X, and Y, are currently or expected to be available in the next 2–4 years [9, 43, 44]. Furthermore, as part of the Gavi 2018 Vaccine Investment Strategy for 2021–2025, the Gavi Board approved, in principle, an expansion of the existing meningococcal vaccine program to support serogroup ACW-containing multivalent meningococcal conjugate vaccines [45]. New formulations of pneumococcal conjugate vaccines are also in development [46, 47]. High-quality meningitis surveillance with laboratory testing to confirm causal pathogen and whole-genome sequencing (WGS) to determine molecular type will be necessary to clearly understand the impact of new multivalent conjugate meningococcal and pneumococcal vaccines on meningitis in Africa. Given this ongoing surveillance need for high-quality data in sub-Saharan Africa, MenAfriNet will continue to support CBS to monitor trends in meningitis disease and epidemics and provide a research platform for the evaluation of new vaccines in key countries. Case-based meningitis surveillance is resource intensive and can be challenging to sustain in countries without well established surveillance and laboratory networks; as such, it is better suited for monitoring disease trends and evaluating vaccine impact than for supporting routine epidemic detection and response. MenAfriNet laboratory confirmed case data complement information from other meningitis surveillance systems in sub-Saharan Africa, such as Integrated Disease Surveillance and Response, Enhanced Meningitis Surveillance, and hospital-based sentinel site surveillance of the WHO’s Invasive Bacterial-Vaccine Preventable Diseases laboratory network [10, 48–50]. Over the next 5 years, MenAfriNet will (1) focus CBS in 2 high-risk countries to improve efficiency, quality, and timeliness of data, (2) leverage MenAfriNet tools and capacity for targeted regional technical assistance, and (3) advance research to optimize current vaccine programs and inform new vaccine strategies.

A paper by Diallo [11] in this supplement describes one MenAfriNet effort to improve timely access to case-based meningitis data. The System for Tracking Epidemiological Data and Laboratory Specimens (STELab) was designed and developed through a collaboration between the Government of Burkina Faso and the West African NGO Davycas International. Developed based on country specifications and MenAfriNet data elements, STELab uses barcodes to ensure that patient data and laboratory testing results can be readily linked and data can be automatically synced to a cloud-based server when an internet connection is available. During a pilot implementation in 2018, STELab improved the frequency, from quarterly to real-time, with which linked epidemiologic data and laboratory testing results were available [11]. Because STELab is implemented nationally in Burkina Faso and Niger, the system will provide more timely data to improve national monitoring of disease trends, outbreak confirmation, support of vaccine program decisions, and evaluation. The STELab system’s novel design and country-driven approach provide real-time meningitis data reporting and specimen tracking for the first time in sub-Saharan Africa; opportunities are being explored to expand this system to include other priority diseases and countries and integrate with other initiatives to improve data access, such as District Health Information Software 2 (DHIS2) [51].

The success of MACV has resulted in an epidemiologic shift in the meningitis belt from the dominance of a single meningococcal serogroup to diverse causes of meningitis disease and epidemics. This shift has necessitated the evolution of surveillance systems toward collection of more patient specimens for laboratory pathogen confirmation, serogrouping/typing, and molecular typing. MenAfriNet CBS aims to collect cerebral spinal fluid specimens from more than 70% of patients with suspected meningitis and transport these specimens to a national reference laboratory for confirmation by polymerase chain reaction (PCR) or culture. MenAfriNet made substantial investments to strengthen national laboratory PCR capacity to confirm pathogens and advance countries’ capability to monitor epidemiologic trends, confirm epidemics, and rapidly implement public health interventions. Strengthening laboratory confirmation capacity, specimen referral, and quality control are critical components of laboratory networks; between 2014 and 2018, MenAfriNet supported 11 trainings to strengthen these critical capacities [12]. Although real-time PCR and culture are effective methods for pathogen detection and assessing the burden of disease, next-generation WGS provides an advanced tool for molecular surveillance of meningococcal and pneumococcal disease. This high-resolution technology enhances existing meningitis surveillance by identifying the emergence of new strains with epidemic potential and monitoring the geographic spread of strains, to inform epidemic preparedness and future vaccination strategies. Although WGS has been implemented in many public health laboratories in high-income countries for surveillance and outbreak investigations, resource-limited countries face challenges related to infrastructure (ie, power fluctuations) and human resources (eg, limited opportunities for training in informatics analysis) when considering this technology. Further innovations in sequencing technology should make WGS more accessible to public health laboratories in resource-limited settings [34]. Until these barriers are bridged, sub-Saharan meningitis belt countries are encouraged to collaborate with global and regional reference laboratories equipped to perform WGS, such as WHO Collaborating Centers and Regional Reference Laboratories [50, 52].

The MenAfriNet platform can be leveraged to strengthen meningitis surveillance beyond the countries where population-based, CBS is directly supported. MenAfriNet tools, procedures, and partnerships were leveraged to strengthen meningitis surveillance capacity and epidemic response in Ghana, Guinea, Liberia, Benin, and Nigeria from 2015 to 2018. Over the next 5 years, MenAfriNet will continue to reinforce surveillance in the African Meningitis Belt through dissemination of best practices and supporting peer-to-peer mentoring. A toolkit of guidelines, protocols, standard operating procedures, and training materials based on the experience and lessons learned during the first 5 years of MenAfriNet has been developed and made publicly available (http://www.menafrinet.org/en-us/Resources/Toolkit). MenAfriNet will continue to support peer-to-peer mentoring and country-to-country technical assistance using the MenAfriNet Toolkit to strengthen data quality in the region. For example, during the Niger NmC epidemic in 2015, MenAfriNet supported Burkina Faso’s deployment of laboratory, surveillance, data management, and immunization experts to support the response. This country-to-country assistance provided surge capacity to improve case detection and laboratory confirmation of NmC and facilitated timely mobilization of reactive vaccination campaigns that reached 1.2 million people [27]. Other examples of country-to-country assistance include Burkina Faso hosting Mali for PCR training, Niger and Burkina Faso deployments to respond to a meningitis epidemic in Togo, and data validation workshops in all 5 MenAfriNet countries. In the future, MenAfriNet aims to continue promoting country-to-country collaboration by supporting countries to identify a roster of deployable country experts. Along with efforts to strengthen regional laboratory capacity to confirm bacterial meningitis pathogens and laboratory quality control/assurance programs, these MenAfriNet-supported activities can help ensure that strategic high-risk countries have adequate surveillance capacity to detect and confirm meningococcal disease and epidemics.

Some questions regarding the effectiveness and duration of protection of bacterial meningitis vaccine programs, including MACV, cannot be answered by surveillance data alone. Papers elsewhere in this supplement highlight research that has been conducted in MenAfriNet-supported countries to address these knowledge gaps [19, 20, 53]. Leveraging the MenAfriNet platform for these studies eliminated logistical barriers and allowed for rapid study implementation with strong country ownership at a lower cost compared with independent research studies (CDC, unpublished data, 2019). For example, cross-sectional meningococcal and pneumococcal nasopharyngeal carriage surveys were conducted in Burkina Faso as part of the strategy to evaluate national immunization policies [54]. Future studies are in development, especially in preparation for future multivalent meningococcal and pneumococcal conjugate vaccines and for evaluating potential changes in the PCV infant immunization schedule. Complementing MenAfriNet meningitis surveillance data with the study of nasopharyngeal carriage will allow assessment of the ability of these new vaccines to induce population-level herd protection.

The initiative to eliminate NmA epidemics in the meningitis belt of sub-Saharan Africa has been an enormous success, yet globally the burden of meningitis remains considerable. The WHO estimated the number of deaths globally from bacterial meningitis in 2015 to be approximately 290 000, with the African region most heavily affected, accounting for 67% of all meningitis deaths [55]. A global roadmap to “Defeat Meningitis by 2030” is being prepared by a WHO-led multiorganization technical task force [56]. The proposed future plans for the meningitis belt to introduce new vaccines and schedules and strengthen surveillance to monitor the impact of these programs on the burden of meningitis in sub-Saharan Africa aligns with the goals of the Defeat Meningitis by 2030 roadmap. After consultation with stakeholders and technical advisory groups, WHO plans to present the global roadmap for adoption at the 72nd World Health Assembly in May 2020.

## CONCLUSIONS

The MenAfriNet consortium has taken an innovative approach to vaccine evaluation through strengthening country meningitis surveillance. This work has been critical for ensuring that quality epidemiologic and laboratory data are available to evaluate progress toward the goal of eliminating epidemics due to NmA in sub-Saharan Africa, to evaluate the impact of new immunization programs such as the PCV program, and to monitor the emergence of bacterial meningitis caused by non-A meningococci and nonvaccine type pneumococci. To meet the goals of WHO’s Defeat Meningitis by 2030 initiative, it will be necessary to address the remaining burden of meningitis in sub-Saharan Africa. MenAfriNet will continue to strengthen surveillance and support research and modeling to inform new multivalent conjugate vaccine programs and increase momentum toward achieving the goal of ending epidemic meningitis as a public health concern in sub-Saharan Africa.

## Notes

***Acknowledgments.*** We thank all MenAfriNet partners, including participating national health systems, health centers, and laboratories. MenAfriNet (www.menafrinet.org) is an international consortium led and implemented by Ministère de la Santé du Burkina Faso, Ministère de la Santé et de l’Hygiène Publique du Mali, Ministère de la Santé Publique du Niger, Ministère de la Santé Publique du Tchad, Ministère de la Santé et de la Protection Sociale du Togo, Agence de Médecine Préventive, Centers for Disease Control and Prevention (CDC), and World Health Organization (WHO), with support and collaboration from CDC Foundation and other international and nongovernmental organizations.

***Financial support.*** This work was funded by the MenAfriNet consortium (www.menafrinet.org) through a grant from the Bill & Melinda Gates Foundation (OPP1084298).

***Potential conflicts of interest.*** All authors: No reported conflicts of interest. All authors have submitted the ICMJE Form for Disclosure of Potential Conflicts of Interest.
